# Promising neuroprotective potential of naringenin against trimethyltin-induced cognitive deficits and hippocampal neurodegeneration in rats

**DOI:** 10.3389/fnins.2025.1567236

**Published:** 2025-05-23

**Authors:** Kimia Faryadras, Ravieh Golchoobian, Saeid Iranzadeh, Mehrdad Roghani

**Affiliations:** ^1^School of Medicine, Shahed University, Tehran, Iran; ^2^Department of Physiology, Cellular and Molecular Biology Research Center, Health Research Institute, Babol University of Medical Sciences, Babol, Iran; ^3^Neurophysiology Research Center, Shahed University, Tehran, Iran

**Keywords:** Alzheimer’s disease, trimethyltin, naringenin, neuroprotection, cognition

## Abstract

**Introduction:**

Learning and memory deficits are clinical characteristics of Alzheimer’s disease (AD), often leading to diminished functionality. The neurotoxicant trimethyltin (TMT) is a valuable research tool for inducing cognitive impairment and hippocampal neurodegeneration and studying AD pathogenesis and treatment. Naringenin is a flavonoid with potential neuroprotective effects. This study sought to investigate the neuroprotective potential of naringenin against hippocampal neurodegeneration induced by TMT neurotoxicity and identify some underlying molecular mechanisms.

**Methods:**

Neurodegeneration was induced through an 8 mg/kg intraperitoneal injection of TMT, followed by oral administration of naringenin (25 and 100 mg/kg) for 21 days. Behavioral assessments, including novel object discrimination (NOD), Y-maze, and passive avoidance tests, were carried out to evaluate cognitive functions. Biochemical assays for oxidative/nitrosative stress, mitochondrial membrane potential (MMP), inflammation, and acetylcholinesterase (AChE) enzyme activity, as well as AD pathology-specific markers, were conducted. To further validate the results, histological assessments of the CA1 hippocampal region using Nissl staining and immunohistochemical identification of 3-nitrotyrosine (3-NT) were performed.

**Results and discussion:**

Naringenin exhibited a dose-dependent inhibition of CA1 neuronal loss and reversed TMT-induced cognitive deficits. It markedly decreased hippocampal levels of malondialdehyde (MDA), nitrite, tumor necrosis factor-alpha (TNFα), and AChE activity while enhancing catalase and superoxide dismutase (SOD) activities, 3-nitrotyrosine (3-NT) immunoreactivity, and MMP. Furthermore, findings demonstrated that naringenin mitigated the TMT-induced elevation in hippocampal levels of AD-specific proteins, including phosphorylated tau (p-tau), amyloid-beta (Aβ), and presenilin 1. Naringenin may be postulated as a promising therapeutic candidate for AD and related neurodegenerative conditions by mitigating oxidative and nitrosative stress, maintaining mitochondrial integrity, decreasing inflammation, and modulating pathways of neurodegeneration.

## 1 Introduction

Alzheimer’s disease (AD) is a chronic and progressive neurodegenerative condition marked by significant cognitive impairments and behavioral deficits, impacting over six million Americans and close to fifty million people worldwide (Kamatham et al., 2024; Rajan et al., 2021). The specific pathological manifestations of AD include intracellular neurofibrillary tangles (NFTs) triggered by hyperphosphorylated tau (p-tau) and extracellular amyloid beta (Aβ) plaque accumulations (Zhang et al., 2021). Aβ and p-tau are responsible for synaptic dysfunction, neuronal death, and cognitive deficits (Rajmohan and Reddy, 2017). Presenilin 1 is a key constituent of the gamma-secretase complex and exerts a vital contribution to the conversion of amyloid-beta precursor protein (APP) into Aβ (Delport and Hewer, 2022). In AD progression, these neurodegenerative pathological mechanisms are intensified by additional non-specific mechanisms such as oxidative and nitrosative stress, mitochondrial dysfunction, and persistent neuroinflammation (Dash et al., 2025; de Oliveira et al., 2021; Guo et al., 2013).

Trimethyltin (TMT), an organotin compound with selective hippocampal targeting, leads to cognitive deficits, accumulation of p-tau and Aβ, oxidative stress, and neuroinflammation in the hippocampus (Babak et al., 2024; Taheri et al., 2024). Therefore, TMT-challenged rodents are a valuable model for assessing prospective therapeutic options that target the molecular pathways involved in AD (Rostami et al., 2022).

Naringenin, 5,7-dihydroxy-2-(4-hydroxyphenyl) chroman-4-one, is a naturally occurring flavonoid belonging to the flavanones subclass and is abundantly found in citrus fruits like grapefruits, oranges, bergamot, and tomatoes. Naringenin demonstrates a wide range of biological properties, encompassing antioxidant, anti-inflammatory, DNA-protective, and immunomodulatory effects. Naringenin has exhibited therapeutic potential across a spectrum of disorders, encompassing cancer, diabetes mellitus, and cardiovascular diseases (Kiran et al., 2017; Mir and Tiku, 2015). Interestingly, the neuroprotective properties of naringenin in diverse neurodegenerative conditions have yielded promising outcomes (Nouri et al., 2019). Experimental studies have shown that naringenin improves cognitive function and neuronal survival through scavenging reactive oxygen species, improving the brain insulin pathway, preserving mitochondrial function, and modulating key signaling pathways involved in neurodegeneration (Ghofrani et al., 2019).

This study aimed to evaluate the therapeutic effectiveness of naringenin against the TMT-induced cognitive deficits and hippocampal neurodegeneration in rats. We utilized behavioral, biochemical, and histological examinations to assess the therapeutic capacity of naringenin in alleviating TMT neurotoxicity and investigating the mechanisms responsible for its neuroprotective effects.

## 2 Materials and methods

### 2.1 Animals

Male Wistar rats (weighing 195–225 *g*) were obtained from the animal center of the Faculty of Biology, SBMU, Tehran. The animals were accommodated in acrylic cages (3–4 Wistar rats per cage) under controlled conditions with a regulated temperature, humidity, and a 12:12-h dark-light cycle. Rodents were provided *ad libitum* accessibility to standardized pellet food and tap water. A one-week acclimation period was observed before initiating experiments. Behavioral tests were conducted in a semi-dark, quiet room between 10:00 and 15:00. The experimental protocol was approved by the Ethics Committee of Shahed University (Approval No. IR.SHAHED.REC.1400.101).

### 2.2 Experimental design

Forty adult rats were randomly allocated to five experimental groups, with each group comprising eight animals. The groups were designated as follows: control; control + naringenin 100; TMT; TMT + naringenin 25; TMT + naringenin 100. The quantity of animals utilized was sourced from a relevant prior investigation (Taheri et al., 2024). The TMT and TMT + naringenin (25 or 100) groups were administered a singular intraperitoneal dose of TMT-chloride (Cat. #sc-301942, Santa Cruz Biotechnology, Inc., United States) at a dosage of 8 mg/kg, dissolved in 0.9% NaCl, as previously determined by Ye et al. (2024). The other groups received an equivalent volume of 0.9% NaCl intraperitoneally. Naringenin (Cat. #N5893, Sigma-Aldrich, United States) was dissolved in cremophor 10%, which served as the vehicle, and was administered daily via oral gavage at doses of 25 mg/kg or 100 mg/kg in the control + naringenin 100 and TMT + naringenin (25 or 100) groups. Administration began 1 h after TMT injection and continued daily for 21 days. The remaining groups received cremophor 10% as a substitute for naringenin (Ghofrani et al., 2019). Supplementary Table 1 shows the effect of cremophor 10% on behavioral indices and hippocampal level of MDA as an index of lipid peroxidation. Our statistical analysis showed no significant difference between the control groups or TMT groups in the presence or absence of the vehicle.

**FIGURE 1:**
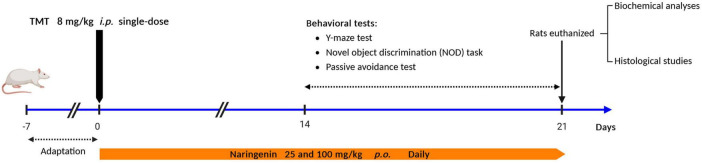
Study design and experimental timeline. TMT was injected intraperitoneally at a dosage of 8 mg/kg to induce neurotoxicity. One hour after TMT injection, naringenin oral administration started at doses of 25 or 100 mg/kg/day for a duration of 3 weeks.

The selection of the naringenin dosage was based on findings from a previous investigation concerning its neuroprotective efficacy in a rat model of dementia (Yang et al., 2014). A detailed schematic representation of the experimental protocol is provided in Figure 1.

Behavioral assessments were conducted at the end of the treatment period by researchers blinded to the experimental groups to ensure an unbiased evaluation. Upon completing the behavioral tests, the animals were administered a high-dose anesthetic combination of ketamine (at 150 mg/kg) and xylazine (at 10 mg/kg), and their brains were subsequently extracted, processed, and preserved for further analysis.

### 2.3 Behavioral tests

#### 2.3.1 Y-maze task

The Y-maze test was utilized to evaluate short-term spatial recognition memory, following the method outlined by Souza et al. (2022). In this task, each rat was randomly introduced into one arm of the maze and permitted to browse without restriction throughout the three arms for eight minutes. The sequence of arm entries was visually recorded. Spontaneous alternation, serving as an indicator of short-term memory, was calculated by dividing the amount of unique overlapping entry sequences by the entire number of arm entries minus 2, then multiplying the result by 100.

#### 2.3.2 Novel object discrimination (NOD) task

The NOD task was employed to evaluate conscious memory of prior experiences, providing a suitable method for analyzing episodic memory. The procedure followed the protocol outlined by Zamani et al. (2019). Briefly, the test involved two consecutive object exploration sessions separated by a 1-h interval. During the initial 5-minute familiarization phase (first session), each rat was presented with two identical objects. In the subsequent 5-minute choice phase (second session), one of the original items was randomly substituted with a new one. Object exploration was characterized by the rat engaging in behaviors such as licking, sniffing, vibrating its vibrissae, or chewing while directing its nose within 2 centimeters of the object. Shortly after each session, the items and surfaces were entirely sanitized using a 70% ethanol solvent to eliminate odor cues. Memory performance was assessed via the discrimination ratio through the formula:


$$\frac{t\,\left[\mathrm{novel}\right]}{t\,\left[\mathrm{total}\,\mathrm{for}\,\mathrm{familiar}\,\mathrm{and}\,\mathrm{novel}\right]}\times 100$$

#### 2.3.3 Passive avoidance task

The relationship between learning and memory in rodents was evaluated using the fear-conditioned (Khajevand-Khazaei et al., 2018). The apparatus comprised two connected chambers, one illuminated and one dim, separated by a guillotine door. An electric foot shock, administered through a grid floor, served as the aversive stimulus. In the acquisition phase, the rats were put into the lit chamber. Subsequent to a 5-minute adaptation period, the examiner opened the guillotine door, and immediately following the rat’s entry into the dim chamber, an electric shock (1 mA, 1 s) was administered. The time spent entering the dim chamber referred to as the initial latency (IL), was recorded. On the following day, animals were placed back in the illuminated chamber to assess memory retention. The duration from positioning in the lighted chamber to the rat’s entry into the dim chamber was noted as the step-through latency (STL), with a cut-off time of 5 min.

### 2.4 Biochemical analyses

#### 2.4.1 Preparation of tissue homogenate

Following behavioral assessments, rats were heavily anesthetized and perfused with physiological saline. Left-side hippocampal tissues were homogenized in ice-cold tromethamine hydrochloride buffer (50 mM, pH 7.6) at a 1:20 (w/v) ratio. Homogenates were *centrifuged* at 5,000 rpm (4°C), and the supernatants were collected for biochemical analyses. Total protein levels were quantified using the bicinchoninic acid (BCA) assay kit (Kiazist, Iran). During the process, proteins catalyze the reduction of bivalent copper to monovalent copper at 55 degrees Celsius for 30 min. Absorbance readings were recorded at 560 nm, with albumin serving as the standard.

#### 2.4.2 Measurement of oxidative/nitrosative stress biomarkers

The hippocampal supernatant amount of malondialdehyde (MDA), as a valid chemical indicator for lipid oxidation (Khaleghi-Mehr et al., 2023), was measured with a commercial kit (KiaZist, Iran). Briefly, the assay entailed adding MDA reagents, including 2-thiobarbituric acid and trichloroacetic acid, to the supernatant. The final mixture was kept at the boiling temperature for 30 min. Following the solution cooling on ice, it was *centrifuged* at 3,000 rpm for 10 min to collect the supernatant, and the absorbance was recorded at 532 nm.

The Griess method was applied to assess hippocampal supernatant nitrite content, as described before (Baluchnejadmojarad and Roghani, 2012). Given the rapid degradation kinetics of nitric oxide (NO), characterized by its brief half-life, this molecule undergoes spontaneous oxidation to yield the stable metabolites nitrate (NO^3–^) and nitrite (NO^2–^). The analytical procedure entails the preliminary reduction of nitrate to nitrite through cadmium-mediated catalysis, followed by a chromogenic reaction. This latter step involves the sequential diazotization of sulfanilamide and subsequent coupling with N-naphthyl ethylenediamine under acidic conditions to generate an azo compound. The resultant chromophore’s absorption intensity was spectrophotometrically determined at a wavelength of 540 nm.

The assessment of superoxide dismutase (SOD) activity utilized commercially available assay kits (Kiazist, Iran). A working solution containing xanthine oxidase and a diluted SOD reagent was prepared, followed by measuring the absorbance at 570 nm. Subsequently, the 50% inhibition and SOD activity levels were quantified and expressed as units per milligram of protein.

To quantify catalase activity, a commercially available assay kit (KiaZist, Iran) containing hydrogen peroxide, methanol, purpald, potassium periodate, and potassium hydroxide solution was employed, followed by measuring absorbance at 550 nm. The results were expressed as catalase activity per milligram of protein using formaldehyde as the standard.

#### 2.4.3 Measurement of mitochondrial membrane potential (MMP)

Measurement of mitochondrial membrane potential (MMP), an established indicator of mitochondrial functional integrity, was evaluated according to previously validated protocols (Ding et al., 2013). The mitochondrial fraction was isolated through differential *centrifugation* of hippocampal supernatant at 10,000 rpm under controlled temporal parameters (15 min). The resultant mitochondria-enriched pellet underwent incubation with rhodamine 123 (Sigma-Aldrich, United States) at a defined concentration of 0.2 μmol/L, maintained at physiological temperature (37°C) for a 5-minute duration. Quantitative assessment of MMP was accomplished via fluorometric analysis, utilizing specific wavelength parameters (λex = 488 nm; λem = 525 nm) in conjunction with a fluorescence-based microplate reader. The fluorescence emission intensity was quantified and reported in arbitrary fluorescence units (AFU).

#### 2.4.4 Quantification of neuroinflammatory and neurodegenerative biomarkers

The concentrations of key biomarkers associated with neuroinflammation, neurodegeneration, and AD pathology were quantified in hippocampal tissue using sandwich enzyme-linked immunosorbent assay (ELISA). Tumor necrosis factor-alpha (TNFα; Cat. #sc-52746, Sigma-Aldrich, United States) was measured as a marker of pro-inflammatory cytokine activity (Qin et al., 2007). P-tau (Cat. #sc-32275, Santa Cruz Biotechnology, Inc., United States) was evaluated as an indicator of tau pathology and microtubule destabilization (Zhang et al., 2021). Aβ Cat. #sc-28365, Santa Cruz Biotechnology, Inc., United States) was assessed as a hallmark of amyloid plaque deposition (Akkaya et al., 2007), while presenilin 1 (Cat. #sc-365495, Santa Cruz Biotechnology, Inc., United States) was measured as an important protein involved in APP processing (Martinez-Feduchi et al., 2024). Absorbance values were recorded using the Synergy HT microplate reader (BioTek Instruments, United States), and final concentrations were calculated according to the standard curves generated for each biomarker. The quantitative data were normalized to total protein content and expressed as picograms per milligram of protein.

#### 2.4.5 Measurement of acetylcholinesterase (AChE) activity

Hippocampal AChE activity was evaluated employing an adapted version of Ellman’s method (Isomae et al., 2003). AChE activity was quantified by measuring the yellow product formed when Ellman’s reagent reacts with thiocholine, which is produced through the enzymatic breakdown of acetylthiocholine. The absorbance at 412 nm was recorded, and the results were expressed as μmol of substrate hydrolyzed per minute per gram of protein.

### 2.5 Histological examination

Following the completion of the behavioral assessments in week 3, coronal sections (5 μm thickness) of the left hippocampus were processed for histological examination, involving Nissl staining with Cresyl violet acetate and immunohistochemical identification of 3-nitrotyrosine (3-NT) after paraffin embedding.

Immunohistochemical procedures were initiated with deparaffinization of tissue sections, followed by rehydration utilizing a Tris-buffered solution (pH 7.4). Antigen unmasking was accomplished via thermal treatment in sodium citrate buffer (pH 6.0) under controlled conditions (10-minute duration). Subsequently, non-specific immunoreactivity was suppressed through incubation with a blocking solution comprising 2.5% bovine serum albumin (Merck, Germany) supplemented with 0.2% Triton X-100 in PBS for 60 min. The specimens were then subjected to primary immunolabeling utilizing mouse monoclonal anti-3-nitrotyrosine antibody (dilution factor 1:125; Cat. #sc-71705, Santa Cruz Biotechnology, United States) under refrigerated conditions (4°C) for 12 h. Secondary immunodetection was performed using horseradish peroxidase-conjugated mouse IgGκ binding protein (dilution factor 1:150; Cat # sc-516102, Santa Cruz Biotechnology, United States). Chromogenic visualization was achieved through the oxidation of diaminobenzidine substrate (DAB; Cat. #sc-209686, Sigma-Aldrich, United States) catalyzed by hydrogen peroxide. Quantitative evaluations were conducted utilizing computer-assisted image analysis software. Immunoreactivity for 3-NT was quantified as integrated optical density (IOD).

To assess neuronal density, pyramidal neurons in the CA1 region of the hippocampus were counted in at least four sections, corresponding to anatomical levels −3.6 to −4.3 mm relative to the bregma. Neuronal counts were conducted within a defined area of 0.1 mm^2^ using an image acquisition and analysis system. Only neurons with well-defined cytoplasmic borders and clearly visible nucleoli were included in the analysis. All histological assessments were conducted on coded slides by a single blinded investigator to ensure unbiased analysis.

### 2.6 Statistical analysis

Statistical analyses were conducted on experimental data, with values expressed as arithmetic means accompanied by their standard errors (mean ± SEM). The Shapiro-Wilk normality test was employed to evaluate the Gaussian distribution of the datasets. Inter-group comparisons were performed using univariate analysis of variance (one-way ANOVA), with subsequent multiple comparisons conducted via Tukey’s honestly significant difference test. Statistical significance was established at a probability threshold of *p* < 0.05. All statistical analyses were executed using the GraphPad Prism software (version 8.4.3).

## 3 Results

### 3.1 Naringenin mitigates TMT-induced behavioral deficits

**FIGURE 2:**
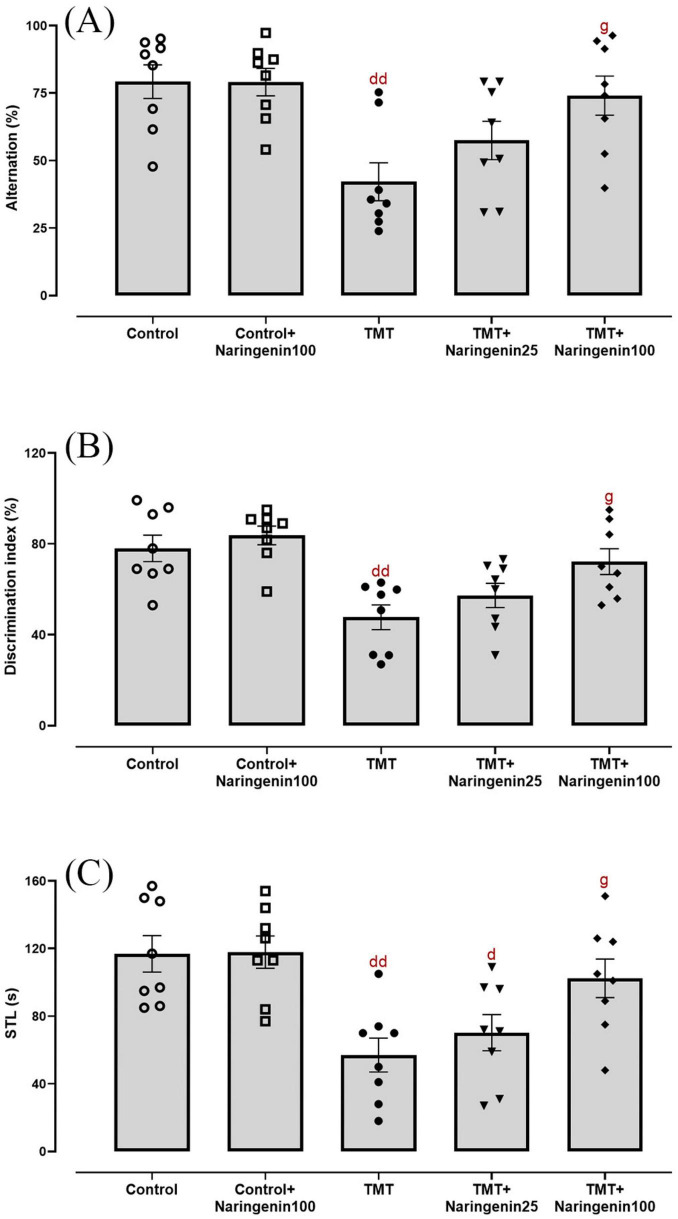
Cognitive and memory performance, demonstrating that naringenin ameliorates TMT-induced deficits. **(A)** Percentage of spontaneous alternation in the Y-maze test, assessing working memory. **(B)** Discrimination index in the NOD task, evaluating recognition memory. **(C)** Step-through latencies (STLs) in the passive avoidance test, measuring fear-associated memory retention. TMT was injected intraperitoneally at a dosage of 8 mg/kg to induce cognitive impairment. One hour after TMT injection, naringenin oral administration started at doses of 25 or 100 mg/kg/day for a duration of 3 weeks. Data are expressed as mean ± SEM (*n* = 8 per group). Statistical analysis was conducted using one-way ANOVA followed by Tukey’s *post hoc* test. d *p* < 0.05, dd *p* < 0.01 vs. control; g *p* < 0.05 vs. TMT group.

Statistical analysis of Y-maze performance, assessing short-term spatial recognition memory, demonstrated significant inter-group variations (F_4, 35_ = 5.043, *p* < 0.01). TMT administration significantly reduced spontaneous alternation percentages compared to controls (*p* < 0.01). Naringenin at a low dose of 25 mg/kg did not ameliorate this deficit (*p* > 0.05). However, treatment with 100 mg/kg naringenin significantly improved spontaneous alternation percentages relative to the TMT group (*p* < 0.05) (Figure 2A).

Evaluation of episodic memory in the NOD task revealed statistically significant between-group differences (*F*_4, 35_ = 7.015, *p* < 0.001). Post-hoc analyses indicated significant impairment in discrimination ratio percentages among TMT-administered rats relative to controls (*p* < 0.01). Administration of 100 mg/kg naringenin successfully preserved this metric (*p* < 0.05), whereas 25 mg/kg naringenin had no significant effect (*p* > 0.05) (Figure 2B).

In the passive avoidance task, inhibitory avoidance was evaluated via IL and STL. In IL time, the treatment groups were not significantly different (data not shown). Concerning STL (Figure 2C), one-way ANOVA indicated a significant inter-group variability (*F*_4, 35_ = 7.031, *p* < 0.001). The TMT-challenged rats (*p* < 0.01) and the TMT-injected rats treated with naringenin at a dose of 25 mg/kg (*p* < 0.05) exhibited a significant reduction in retention and recall ability in this test as compared to control, as evidenced by a shorter STL. However, naringenin at 100 mg/kg significantly improved STL (*p* < 0.05) compared to the TMT group. Notably, naringenin administration at 100 mg/kg did not produce significant changes in any of the behavioral tests (Y-maze, NOD, and passive avoidance tests) conducted on the control + naringenin 100 group, with all *p*-values > 0.05 (Figure 2).

### 3.2 Naringenin reduces TMT-induced hippocampal oxidative/nitrosative stress, mitochondrial dysfunction, and inflammation

**FIGURE 3:**
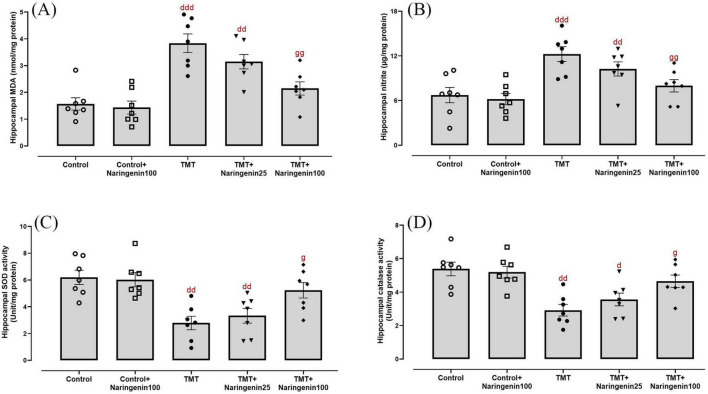
Oxidative and nitrosative stress markers in the hippocampus, indicating that naringenin mitigates TMT-induced oxidative damage. **(A)** Malondialdehyde (MDA) levels, indicating lipid peroxidation. **(B)** Nitrite levels, representing nitric oxide metabolism. **(C)** Superoxide dismutase (SOD) activity and **(D)** catalase activity, both serving as key antioxidant defense markers. TMT was injected intraperitoneally at a dosage of 8 mg/kg to induce neurotoxicity. One hour after TMT injection, naringenin oral administration started at doses of 25 or 100 mg/kg/day for a duration of 3 weeks. Data are expressed as mean ± SEM (*n* = 7 per group). Statistical analysis was conducted using one-way ANOVA followed by Tukey’s *post hoc* test. d *p* < 0.05, dd *p* < 0.01, ddd *p* < 0.001 vs. control; g *p* < 0.05, gg *p* < 0.01 vs. TMT group.

Quantitative assessment of oxidative/nitrosative stress parameters revealed significant variations in hippocampal MDA concentrations (Figure 3A; *F*_4, 30_ = 14.12, *p* < 0.001), nitrite levels (Figure 3B; *F*_4, 30_ = 10.65, *p* < 0.001), SOD activity (Figure 3C; *F*_4, 30_ = 8.56, *p* < 0.001), and catalase activity (Figure 3D; *F*_4, 30_ = 6.69, *p* < 0.001). Naringenin (100 mg/kg) treatment of the control animals did not exert any statistically significant change in the hippocampal levels of oxidative/nitrosative markers (p > 0.05). The TMT and TMT + naringenin 25 groups exhibited significantly higher levels of hippocampal MDA (*p* < 0.001 and *p* < 0.01, respectively) and nitrite (*p* < 0.001 and *p* < 0.01, respectively), as well as lower SOD (*p* < 0.01 for both) and catalase activity (*p* < 0.01 and *p* < 0.05, respectively) compared to the control rats. However, treatment with naringenin at a dose of 100 mg/kg in TMT-injected rats significantly reduced MDA (*p* < 0.01) and nitrite (*p* < 0.01) levels and improved SOD (*p* < 0.05) and catalase activity (*p* < 0.05) compared to the TMT group (Figure 3).

**FIGURE 4:**
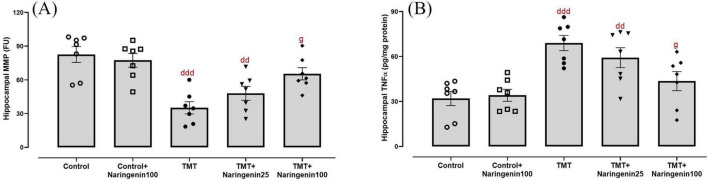
Mitochondrial integrity and neuroinflammatory responses in the hippocampus, showing that naringenin attenuates TMT-induced mitochondrial dysfunction and neuroinflammation. **(A)** MMP levels, indicative of mitochondrial integrity. **(B)** Tumor necrosis factor-alpha (TNFα) levels, reflecting neuroinflammatory responses. TMT was injected intraperitoneally at a dosage of 8 mg/kg to induce neurotoxicity. One hour after TMT injection, naringenin oral administration started at doses of 25 or 100 mg/kg/day for a duration of 3 weeks. Data are expressed as mean ± SEM (*n* = 7 per group). Statistical analysis was conducted using one-way ANOVA followed by Tukey’s *post hoc* test. dd *p* < 0.01, ddd *p* < 0.001 vs. control; g *p* < 0.05 vs. TMT group.

Mitochondrial dysfunction was investigated through the biochemical assessment of MMP levels in hippocampal tissue. The data demonstrated significant between-group differences (Figure 4A; *F*_4,30_ = 8.121, *p* < 0.001). Treatment with naringenin at a dose of 100 mg/kg in the control animals did not result in a statistically significant alteration in hippocampal MMP levels (p > 0.05). Both the TMT and TMT + naringenin 25 groups exhibited significantly reduced MMP levels (with *p*-values < 0.001 and < 0.01, respectively) relative to the control rats. In contrast, the administration of a higher dosage of naringenin (100 mg/kg) in TMT-injected rats effectively increased MMP levels significantly (*p* < 0.05).

As shown in Figure 4B, hippocampal TNFα levels, a pro-inflammatory cytokine, exhibited significant differences between the study groups (*F*_4, 30_ = 8.606, *p* < 0.001). *Post hoc* test analysis demonstrated elevated TNFα levels in the TMT (*p* < 0.001) and the TMT + naringenin 25 groups (*p* < 0.01) compared to the controls. However, administering naringenin at a dose of 100 mg/kg to the TMT group significantly reduced this inflammation marker (*p* < 0.05) relative to the TMT group. Additionally, TNFα levels in control + naringenin 100 rats did not differ significantly from those in the control group (*p* > 0.05).

### 3.3 Naringenin reverses TMT-induced hippocampal levels of neurodegeneration biomarkers and AChE activity

**FIGURE 5:**
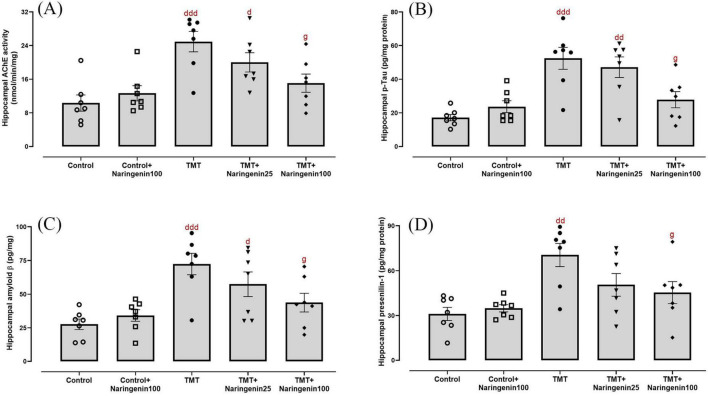
Cholinergic dysfunction and AD-related markers in the hippocampus, demonstrating that naringenin alleviates TMT-induced cholinergic impairment and AD-associated pathology. **(A)** Acetylcholinesterase (AChE) activity, assessing cholinergic functioning. **(B)** Phosphorylated tau (p-Tau), a key marker of tau hyperphosphorylation associated with neurodegeneration, **(C)** amyloid β, a hallmark of AD pathology, and **(D)** presenilin 1, a regulator of amyloid precursor protein processing and amyloid β formation, levels. TMT was injected intraperitoneally at a dosage of 8 mg/kg to induce neurotoxicity. One hour after TMT injection, naringenin oral administration started at doses of 25 or 100 mg/kg/day for a duration of 3 weeks. Data are expressed as mean ± SEM (*n* = 7 per group). Statistical analysis was conducted using one-way ANOVA followed by Tukey’s *post hoc* test. d *p* < 0.05, dd *p* < 0.01, ddd *p* < 0.001 vs. control; g *p* < 0.05 vs. TMT group.

One-way ANOVA revealed significant effects of TMT injection and naringenin treatment on the hippocampal levels of AChE activity (*F*_4, 30_ = 6.93, *p* < 0.001) (Figure 5A) and AD-associated biomarkers, including p-Tau (*F*_4, 30_ = 7.546, *p* < 0.001) (Figure 5B), Aβ (*F*_4, 30_ = 7.703, *p* < 0.001) (Figure 5C), and presenilin 1 (*F*_4, 30_ = 5.985, *p* < 0.001) (Figure 5D). Subsequent analyses revealed significant elevations in AChE activity (*p* < 0.001), p-Tau (*p* < 0.001), Aβ (*p* < 0.001), and presenilin 1 (*p* < 0.01) in TMT-administered rats, relative to controls. Moreover, the TMT + naringenin 25 group exhibited increased levels of AChE activity, p-Tau, and Aβ in comparison to the control group, with corresponding *p*-values of < 0.05, < 0.01, and < 0.05, respectively. More interestingly, the administration of naringenin at a dosage of 100 mg/kg notably reversed the heightened levels of AChE activity (*p* < 0.05), p-Tau (*p* < 0.05), Aβ (*p* < 0.05), and presenilin 1 (*p* < 0.05) induced by TMT. Additionally, naringenin (100 mg/kg) alone did not yield a substantial impact on these parameters (all with *p* > 0.05).

### 3.4 Naringenin prevents TMT-induced hippocampal CA1 neurodegeneration and nitrosative stress

**FIGURE 6:**
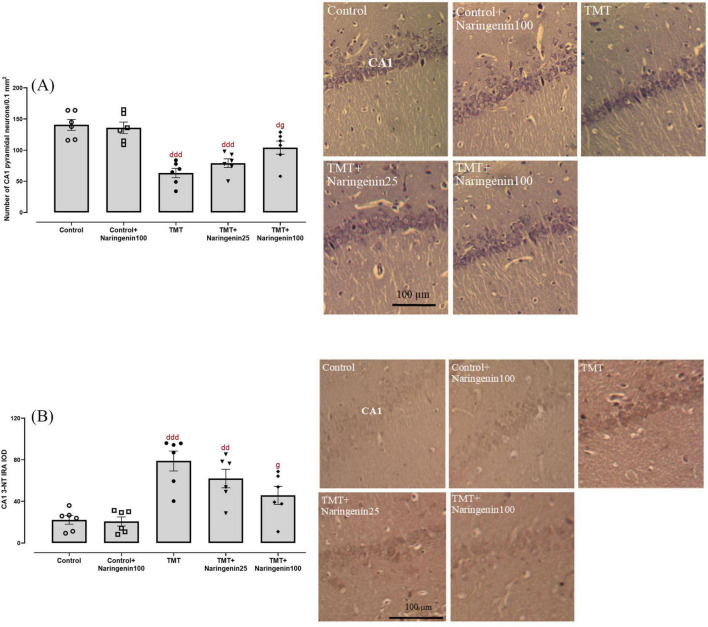
Histopathological analysis of hippocampal CA1 neurons, showing that naringenin prevents TMT-induced neuronal loss and oxidative damage. **(A)** Quantitative analysis of neuronal density in the CA1 region using Nissl staining. **(B)** 3-nitrotyrosine (3-NT) immunoreactivity, assessing protein nitration as an indicator of nitrosative stress. Representative photomicrographs illustrate histological changes across experimental groups. TMT was injected intraperitoneally at a dosage of 8 mg/kg to induce neurotoxicity. One hour after TMT injection, naringenin oral administration started at doses of 25 or 100 mg/kg/day for a duration of 3 weeks. Data are expressed as mean ± SEM (*n* = 6 per group). Statistical analysis was conducted using one-way ANOVA followed by Tukey’s *post hoc* test. d *p* < 0.05, dd *p* < 0.01, ddd *p* < 0.001 vs. control; g *p* < 0.05 vs. TMT group.

Regarding the quantitative assessment of neuronal density in the CA1 region, statistical analysis revealed significant inter-group variations (*F*_4, 25_ = 17.30, *p* < 0.001). Post-hoc analyses demonstrated significant neuronal depletion in the TMT-treated (*p* < 0.001), TMT + Naringenin 25 mg/kg (*p* < 0.001), and TMT + Naringenin 100 mg/kg rats (*p* < 0.05) relative to controls. Notably, administration of naringenin at 100 mg/kg resulted in significant preservation of Nissl-positive neuronal populations compared to TMT-only subjected animals (*p* < 0.05) (Figure 6A).

Assessment of nitrosative stress, conducted via immunohistochemical detection of 3-NT moieties, demonstrated significant between-group variations (*F*_4, 25_ = 10.94, *p* < 0.001). Densitometric analysis revealed significantly elevated 3-NT immunoreactivity within the CA1 hippocampal subfield of TMT-injected rats compared to controls (*p* < 0.001). A similarly substantial rise at a lower magnitude was observed in the TMT + naringenin 25 group (*p* < 0.01). Conversely, specimens from subjects receiving TMT + naringenin at 100 mg/kg demonstrated significantly reduced 3-NT immunoreactivity compared to the TMT-challenged rats (*p* < 0.05) (Figure 6B).

## 4 Discussion

This study aimed to investigate the protective effects of naringenin against TMT-induced neurotoxicity, focusing on molecular mechanisms. Our findings demonstrated that a three-week naringenin treatment regimen effectively alleviated cognitive impairments caused by TMT intraperitoneal injection, potentially through naringenin’s antioxidant, anti-inflammatory, mitochondrial-protective, and neurodegeneration-modulating properties.

Our behavioral assessments indicated that TMT administration led to substantial deficits in learning and memory. Behavioral deficits have been reported following TMT administration, as evidenced by reduced short-term spatial recognition memory (Kim et al., 2021), passive avoidance fear memory (Liu et al., 2023), and working memory (Yoshikawa et al., 2019), consistent with the findings of the current study. Treatment with naringenin reversed these deficits and also offered more evidence to support its neuroprotective role in models of cognitive dysfunction, as documented in prior studies (Rai et al., 2024a; Rai et al., 2024b).

Oxidative and nitrosative stress are key contributors to the pathophysiology of neurodegenerative diseases and are closely associated with neuronal damage, mitochondrial dysfunction, and cognitive decline (Butterfield and Boyd-Kimball, 2020). The study findings revealed that TMT exposure elevated hippocampal levels of nitrite and MDA while reducing antioxidant enzymes, including SOD and catalase. These results align with previous studies on oxidative stress in TMT-exposed animals and neuronal cultures (Song et al., 2021; Thong-Asa et al., 2020). Moreover, TMT increased 3-NT immunoreactivity, a marker of nitrosative stress and protein oxidation, exacerbating protein dysfunction and aggregation. 3-NT and protein carbonyl levels have been shown to be elevated in the frontal cortex of AD and mild cognitive impairment (MCI) patients (Arslan et al., 2020). In our study, naringenin treatment mitigated these effects by reducing nitrite and MDA levels, decreasing 3-NT immunoreactivity, and restoring antioxidant enzyme activity, consistent with previous studies (Zaidun et al., 2018). The antioxidant properties of flavonoids arise from their ability to directly scavenge ROS and NOS and chelate transition metals, thereby preventing radical formation (Horniblow et al., 2017; Symonowicz and Kolanek, 2012). Moreover, these natural agents upregulate endogenous antioxidant defenses and modulate redox-sensitive signaling pathways (Calderaro et al., 2022). This complex mechanism suggests the potential of naringenin in ameliorating the biochemical pathways associated with oxidative/nitrosative stress and consequent neural damage in toxicological and neurodegenerative models (Al-Ghamdi et al., 2021; Khajevand-Khazaei et al., 2018). The absence of a substantial disparity between the control + naringenin 100 group and the control group concerning oxidative stress biomarkers indicates that naringenin primarily exerts its effects in response to oxidative stress rather than modifying redox homeostasis in unstressed conditions. This is consistent with previous research showing that several polyphenolic compounds, including naringenin, specifically increase endogenous antioxidant responses during oxidative stress without inducing variations in antioxidant enzyme levels under physiological conditions (Hosseini et al., 2022; Saati, 2025). Furthermore, flavonoids may activate the nuclear factor erythroid 2-related factor 2 (Nrf2) pathway, which is upregulated in response to oxidative insults, thereby contributing to their selective antioxidant action in pathological states (Mendonca and Soliman, 2020; Shi et al., 2023). Future studies involving direct assays of Nrf2 activation and other redox-sensitive signaling pathways would provide further mechanistic insights into the role of naringenin in modulating oxidative stress responses.

Mitochondrial dysfunction, as one of the non-specific characteristic features of AD and other neurodegenerative conditions, is frequently initiated by oxidative stress and impaired energy metabolism (Bhatti et al., 2017; Swerdlow et al., 2010). In this investigation, exposure to TMT resulted in a notable decrease in MMP, suggesting compromised mitochondrial integrity. Naringenin averted the decline in MMP caused by TMT, demonstrating its capacity to maintain mitochondrial function. These results are consistent with research indicating that naringin, a natural flavanone glycoside, safeguards mitochondrial bioenergetics, preserves calcium homeostasis, lowers heme oxygenase-1 levels, and inhibits apoptosis in an Aβ rat model of AD (Bhatti et al., 2017). Moreover, in the case of ischemia-reperfusion injury model of rats, Gaur et al. showed that naringin reduced glutathione levels, enhanced catalase activity, and normalized mitochondrial enzyme functions in the cerebellum, striatum, and cortex (Gaur et al., 2009).

In this study, significantly elevated levels of TNFα were observed in the hippocampus of the TMT-challenged group, a response that was dose-dependently reduced by naringenin treatment. The susceptibility of the hippocampus to inflammatory injuries is attributed to its high concentration of receptors for inflammatory mediators (Yirmiya and Goshen, 2011). The activation of microglia and astrocytes plays a significant role in the inflammatory response within the hippocampus in TMT-induced neurotoxicity, which correlates closely with neuronal degeneration and behavioral abnormalities (Corvino et al., 2013; Dragić et al., 2021; Kim et al., 2014). The reduction of TNFα by naringenin is presumed to occur through the modulation of signaling pathways linked to microglial activation, indicating its potential to interfere with the neuroinflammatory cascades responsible for hippocampal injury and cognitive impairments (Chen et al., 2019). Furthermore, studies have demonstrated that pharmacological suppression of TNFα can minimize cognitive deficits (Shin et al., 2014). These findings align with earlier research illustrating the efficacy of naringenin in ameliorating cognitive impairments through its anti-inflammatory properties (Khajevand-Khazaei et al., 2018; Zhang et al., 2022).

The accumulation of p-tau, Aβ plaques, and presenilin 1 has been shown to increase in both the brains of AD patients (Yu and Wu, 2021) and TMT-induced AD-like phenotypes of rodents (Park et al., 2021). In this investigation, exposure to TMT markedly elevated the levels of these biomarkers in the hippocampus, mirroring the neurotoxic pathways observed in AD. Treatment with naringenin decreased hippocampal p-tau, Aβ, and presenilin 1 levels, indicating its regulatory impact on crucial neurodegenerative pathways. The findings of this study align with prior research suggesting that naringenin inhibits p-tau formation through downregulating glycogen synthase kinase-3β (GSK-3β) (Yang et al., 2014). Moreover, naringin, which is converted into naringenin in the liver by the naringinase enzyme (Ribeiro, 2011), has demonstrated potency in inhibiting cyclin-dependent kinase 5 (CDK5), a significant regulator of tau phosphorylation in rats (Meng et al., 2021). Additionally, *in vitro* studies have shown naringenin’s ability to inhibit β-secretase 1 (BACE1). Through the reduction of BACE1 expression and activity, naringenin diminishes APP processing and consequent Aβ production, alleviating the amyloidogenic pathway (Lee et al., 2018). Our findings regarding the hippocampal levels of presenilin 1 are notably innovative, given the limited research on the influence of naringenin on presenilin 1, an integral component of the gamma-secretase proteolytic complex responsible for cleaving APP into Aβ peptides in familial AD. Elevated levels of presenilin 1 correlate with amplified Aβ production and greater neurotoxic effects (Delport and Hewer, 2022). The observed decrease in the hippocampal presenilin 1 levels following naringenin treatment indicates a previously uninvestigated mechanism through which naringenin could regulate Aβ production and alleviate AD-related pathology.

Cholinergic dysfunction is strongly associated with cognitive impairment, as observed following TMT administration and in animal models of AD (Huang et al., 2022). Increased AChE activity and production are pivotal components of this dysfunction, contributing to Aβ peptide deposition, enhanced neurotoxicity, and cognitive decline (Salari et al., 2024). AChE interacts directly with presenilin 1, resulting in elevated Aβ levels and exacerbated cognitive impairments (Campanari et al., 2014; Cortés-Gómez et al., 2023). Additionally, abnormal cholinergic system changes provoke p-tau formation, neuronal apoptosis, and neuroinflammation, further exacerbating neurodegeneration (Chen et al., 2022). In this study, TMT-induced neurotoxicity exhibited a correlation with elevated hippocampal AChE activity, possibly attributable to its interactions with presenilin 1, Aβ, and p-tau proteins. Enhanced AChE activity can also originate from degenerating cholinergic neurons releasing excess AChE, a hypothesis substantiated by existing literature. Interestingly, both Aβ and p-tau can influence AChE expression, suggesting a bidirectional interaction that amplifies neurodegeneration (García-Ayllón et al., 2011). Naringenin administration at 100 mg/kg markedly reduced hippocampal AChE activity, maintaining cholinergic neurotransmission. This aligns with evidence that naringenin mitigates Aβ-induced cognitive impairment by AChE activity (Khajevand-Khazaei et al., 2018).

Histological analysis further corroborated these findings by revealing substantial neuronal loss in the hippocampal CA1 region of the TMT group, consistent with previous reports of tremendous decreases in the number of pyramidal cells following TMT administration (Mataram et al., 2021). Naringenin treatment significantly preserved neuronal density in the CA1 region, highlighting its protective effects against TMT-induced hippocampal neurodegeneration. This aligns with findings from Tayyab et al., who reported similar neuroprotective effects of naringenin in a rat model of chronic mild stress, where it mitigated CA1 cellular morphological anomalies and cell depletion (Tayyab et al., 2019).

The safety profile of naringenin has been widely studied, with multiple reports supporting its high tolerability even at doses exceeding 100 mg/kg (Bellavite, 2023; Younes et al., 2024). In the present study, the absence of significant alterations in oxidative stress, inflammatory markers, and AD-related proteins in the control group receiving naringenin suggests that it does not induce significant adverse/beneficial biochemical or histopathological changes under normal physiological conditions. This aligns with prior findings demonstrating that flavonoids primarily exert their protective effects under pathological conditions rather than modulating homeostatic parameters in unstressed models (Ghofrani et al., 2019; Ikram et al., 2019; Song et al., 2024). These findings reinforce the notion that naringenin functions as a conditionally active neuroprotective agent, specifically targeting pathological alterations without disrupting normal homeostasis.

Animal models, including transgenic and neurotoxicant-induced approaches, have yet to fully replicate the pathological complexity of AD (Pádua et al., 2024). Among these, TMT is a well-established model for studying hippocampus-specific neurodegeneration and cognitive deficits. While not an AD-specific neurotoxin, TMT induces oxidative stress, inflammation, apoptosis, and autophagy, contributing to neurodegenerative processes and cognitive impairment. However, TMT does not accurately replicate the cognitive and biochemical changes associated with AD or the behavioral modifications like weight loss, irritability, hypothermia, and tremors (Lee et al., 2016; More et al., 2016; Pompili et al., 2020).

While this study provides robust evidence of naringenin’s neuroprotective mechanisms, several limitations warrant consideration. The long-term effects of naringenin treatment remain unclear, as does its efficacy in advanced stages of neurodegeneration. Additionally, alternative routes of administration and dose optimization were not explored, which could impact its clinical translation. Furthermore, although our current methodological approach, which includes behavioral, biochemical, and histological analyses, provides a comprehensive evaluation of mechanisms of naringenin neuroprotective effects, incorporating additional techniques such as Western blotting and immunofluorescence staining, as well as analyses of signaling pathways and gene expression changes for the analyzed factors could further strengthen the findings. Future studies should address these gaps and investigate naringenin’s potential in combination therapies targeting multiple AD-related pathways.

This study demonstrates the multifaceted neuroprotective effects of naringenin against TMT-induced cognitive impairment and neurotoxicity. By attenuating oxidative and nitrosative stress, preserving mitochondrial function, reducing inflammation, modulating neurodegeneration pathways, and protecting cholinergic function, naringenin emerges as a promising therapeutic candidate for AD and related neurodegenerative disorders.

## Data Availability

The raw data supporting the conclusions of this article will be made available by the authors, without undue reservation.
