# Supplementary material for: Promising neuroprotective potential of naringenin against trimethyltin-induced cognitive deficits and hippocampal neurodegeneration in rats
Source: Front Neurosci. 2025 May 23;19:1567236. doi: 10.3389/fnins.2025.1567236 (PMC12141333; doi:10.3389/fnins.2025.1567236)
Supplement: Supplementary file 1 [file Table_1.docx]

Supplementary Material

# Supplementary Figures and Tables

## Supplementary Table

**Supplementary Table 1.** The effect of cremophor 10% on behavioral indices and hippocampal level of MDA as an index of lipid peroxidation^a^.

|  | **Control-Cremophor10%** | **Control+ Cremophor10%** | **P value** | **TMT-Cremophor10%** | **TMT+ Cremophor10%** | **P value** |
| --- | --- | --- | --- | --- | --- | --- |
| Y-maze alternation (%)  n=8/group | 76.35±4.85 | 79.24±6.21 | > 0.05^b^ | 45.98±6.83 | 42.16±7.02 | > 0.05^b^ |
| Novel object recognition (%)  n=8/group | 80.35±5.71 | 78.03±5.83 | > 0.05^b^ | 45.91±4.37 | 47.69±5.41 | > 0.05^b^ |
| Passive avoidance STL (s)  n=8/group | 112.9±7.52 | 116.5±10.73 | > 0.05^b^ | 54.63±7.62 | 57.03±9.94 | > 0.05^b^ |
| MDA (nmol/mg) n=7/group | 1.74±0.21 | 1.57±0.22 | > 0.05^b^ | 4.03±0.40 | 3.82±0.34 | > 0.05^b^ |

^a^ Values are expressed as mean ± SEM.

^b^ obtained by independent t-test.
